# Vision Difficulty and Engagement in Care Among Aging Men Living With HIV

**DOI:** 10.1093/geroni/igab046.669

**Published:** 2021-12-17

**Authors:** Alison Abraham, Weiqun Tong, Valentina Stosor, Mackey R Friedman, Roger Detels, Michael Plankey

**Affiliations:** 1 University of Colorado--Denver, Aurora, Colorado, United States; 2 Johns Hopkins University, Baltimore, Maryland, United States; 3 Northwestern University Feinberg School of Medicine, Chicago, Illinois, United States; 4 University of Pittsburgh, Pittsburgh, Pennsylvania, United States; 5 UCLA School of Public Health, Los Angelas, California, United States; 6 Georgetown University, Washington, District of Columbia, United States

## Abstract

For aging adults living with HIV (AALH) who have complex medical care needs, vision impairment may be an added burden that may lead individuals to disengage from their own medical care. We examined the relationships of self-reported vision difficulty with indicators of care engagement: 1) adherence to HIV antiretroviral therapy (ART; defined as taking ≥95% of medications); 2) self-reported avoidance of medical care; 3) self-reported tendency to ask a doctor questions about care (> 2 questions at a medical visit). A modified version of the National Eye Institute vision function questionnaire was administered at three semi-annual visits (from October 2017 to April 2018) to assess difficulty performing vision-dependent tasks (no, a little, moderate to extreme difficulty). We included 1063 AALH participants (median age 60 years, 24% Black). Data were analyzed using repeated measures logistic regression with generalized estimating equations adjusted for fixed race, and at visit values for age, education level, depressive symptoms, alcohol use, and smoking status. Compared to no vision difficulty, those reporting moderate to extreme vision difficulty on at least one task (18%) had 1.95 times higher odds (95% CI: 1.36, 2.79) of having less than optimal ART adherence and 1.92 times higher odds [95% CI: 1.06, 3.47]) of avoiding necessary medical care, but 1.6 times higher odds [95%CI: 0.93, 2.72] of asking more questions. These findings suggest that vision impairment plays a role in medical care engagement among older adults living with HIV, and may contribute to poorer management of HIV and chronic comorbidities.

